# An Electrostatic Self-Excited Resonator with Pre-Tension/Pre-Compression Constraint for Active Rotation Control

**DOI:** 10.3390/mi12060650

**Published:** 2021-06-01

**Authors:** Ruide Yun, Yangsheng Zhu, Zhiwei Liu, Jianmei Huang, Xiaojun Yan, Mingjing Qi

**Affiliations:** 1 School of Energy and Power Engineering, Beihang University, Beijing 100191, China; ruideyun@buaa.edu.cn (R.Y.); zhuys_cn@buaa.edu.cn (Y.Z.); liuzhiwei1991@163.com (Z.L.); jmhuang@buaa.edu.cn (J.H.); yanxiaojun@buaa.edu.cn (X.Y.); 2 Collaborative Innovation Center of Advanced Aero-Engine, Beijing 100191, China; 3 National Key Laboratory of Science and Technology on Aero-Engine Aero-Thermodynamics, Beijing 100191, China; 4 Beijing Key Laboratory of Aero-Engine Structure and Strength, Beijing 100191, China

**Keywords:** electrostatic self-excited resonator, pre-tension/pre-compression constraint, variable velocity-position characteristics, active rotation control

## Abstract

We report a novel electrostatic self-excited resonator driven by DC voltage that achieves variable velocity-position characteristics via applying the pre-tension/pre-compression constraint. The resonator consists of a simply supported micro-beam, two plate electrodes, and two adjustable constraint bases, and it can be under pre-compression or pre-tension constraint by adjusting the distance *L* between two constraint bases (when beam length *l* > *L*, the resonator is under pre-compression and when *l* < *L*, it is under pre-tension). The oscillating velocity of the beam reaches the maximum value in the position around electrodes under the pre-compression constraint and reaches the maximum value in the middle position between two electrodes under the pre-tension condition. By changing the constraint of the microbeam, the position of the maximum velocity output of the oscillating beam can be controlled. The electrostatic self-excited resonator with a simple constraint structure under DC voltage has great potential in the field of propulsion of micro-robots, such as active rotation control of flapping wings.

## 1. Introduction and Background

Insects in nature have a strong flying ability. They can freely and easily take off, land, and hover by flapping their wings controlled by muscles in one flapping cycle. Generally, the wings’ flapping velocity and attack angle are variable. For example, wings flap quickly when their attack angles are positive and slowly when the attack angles are negative [1,2,3]. The key aerodynamic mechanism that can effectively increase the lift of the flapping wing is the Kramer effect [2,4], which can enhance lift generation when the wings undergo rapid rotation about the spanwise axis at the end of a stroke. This aerodynamic effect needs the flapping wing to have variable velocity-position characteristics to generate effective lift force [4,5].

However, it is difficult for the traditional drive mechanisms (such as magnetic motor [6,7,8], piezoelectric [9], and electrostatic [10,11,12]) to achieve variable velocity-position characteristics of flapping wings like insects [13,14]. These drive devices usually work in simple, harmonic motion with invariable, velocity-position characteristics. In the bionic robot study, there are many ways to achieve variable velocity output control for lift generation. For motor-drive devices, previous work used elastic elements [1,15] at the root of the wing and spar to change the peak torque of the motor and designed compliant transmission mechanisms [16] to control the oscillating velocity of the drive devices. For electrostatic and piezoelectric driving devices, the elastic hinge [17,18] is often used to change the oscillating velocity output for high lift generation.

Implementing extra mechanical structure [6,19,20] could be a way to obtain variable velocity-position characteristics for lift generation, but the extra structure will increase the complexity and weight of the device. In this paper, we use a novel way to achieve the variable velocity-position characteristics without using the extra mechanic structure. First, the oscillation process of a single beam resonator is studied. The maximum oscillating velocity output position of the beam can be changed from the middle position between two electrodes to the position near the electrodes by changing the constraint condition of the beam from pre-tension to pre-compression. The dynamic model is built to simulate the oscillation process of the beam, and experiments are conducted to verify the simulation model. Further, a novel double-beam resonator is presented by combining two beams with two different constraints (pre-stretched and pre-compressed) to achieve advanced rotations of flapping wings, which is attractive in the field of microrobots.

## 2. Materials and Methods

### 2.1. Structure and Principle

**Figure 1:**
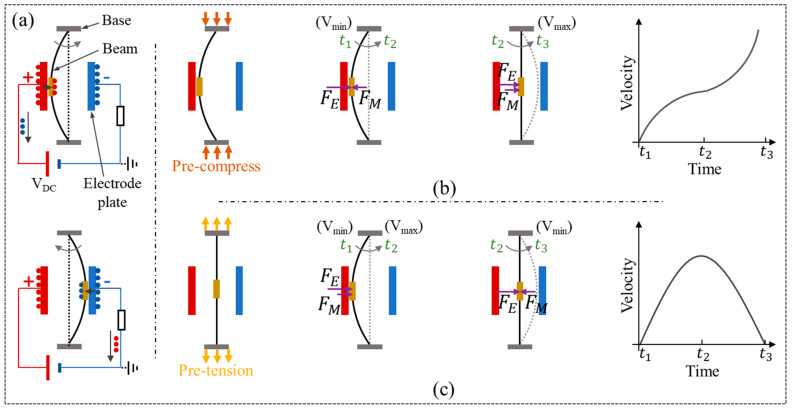
Structure, working principle, and force analysis of the electrostatic self-excited resonator with pre-compression and pre-tension constraints: (**a**) The charge flows in the circuit when the beam makes contact with the positive and negative electrode. (**b**) The force analysis of the microbeam under pre-compression constraint. (**c**) The force analysis of the microbeam under pre-tension constrain.

The structure and working principle of the electrostatic self-excited resonator (single beam prototype) are shown in Figure 1a. A microbeam is assembled between two constraint bases and two electrodes. The beam will start a self-excited oscillation between the positive and negative electrodes with electric charge transfer between two electrodes [10]. Self-excited means that the beam can start and sustain an oscillation under DC voltage controlled by itself; the oscillating frequency is related to its natural frequency but not the frequency of the driving signal. In the oscillation, the resonator is subjected to the combined action of electrostatic force $F_{E}$ and elastic restoring force $F_{M}$. As shown in Figure 1b,c, the application of the pre-compression or pre-tension constraint will change the action of the elastic restoring force $F_{M}$ and allow the resonator to obtain different velocity-position characteristics.

#### 2.1.1. Electrostatic Force

In this paper, the variable equivalent capacitance is used to calculate the electrostatic force, which is expressed as follows:

(1) $$F_{E}=\left(C_{a}^{\prime }-\Delta C_{a}\right)\frac{U^{2}}{d}$$

where $C_{a}^{\prime }$ is the inclined capacitor formed by microbeam and plate electrode, $\Delta C_{a}$ is a part of the capacitance formed by positive and negative plate electrodes, U is powering DC voltage, and d is the vertical distance between two electrodes. Detailed calculation of the parameters for the electrostatic force can be seen in Appendix A.

#### 2.1.2. Quasi-Steady Analysis

**Figure 2:**
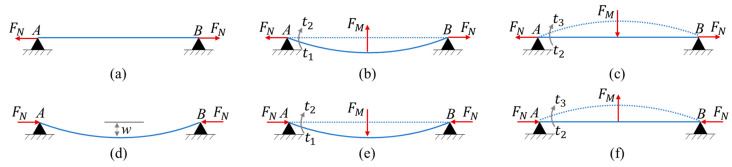
Quasi-steady force analysis of the self-excited resonator with pre-compression and pre-tension constraints for the shift of stable position. (**a**–**c**) Vibration process of the beam under pre-tension; (**d**–**f**) Vibration process of the beam under pre-compression. (**a**,**d**) are the stable position; (**b**,**e**) are the dynamic process from $t_{1}$ to $t_{2}$; (**c**,**f**) are the dynamic process from $t_{2}$ to $t_{3}$. The solid lines mean the position at current moment, and the dotted lines represent the position in the next.

The shift of the stable position will be discussed in this section when the constraint of the beam is changed from pre-tension to pre-compression. In the initial state, as shown in Figure 2a,d, the beam keeps a line when it is under the tension force $F_{N}$, but the beam keeps a curve when it is under the buckling force $F_{N}$. When the beam moves from the same position, the restoring force $F_{M}$ will drive the beam when beam is under pre-tension (in Figure 2b) but will hinder the beam when beam is under pre-compression (in Figure 2e). However, when the beam moves from $t_{2}$ to $t_{3}$, the $F_{M}$ will hinder the beam when beam is under pre-tension, as shown in Figure 2c, and will drive the beam when beam is under pre-compression, as shown in Figure 2f. In one motion cycle, the action of the force $F_{M}$ occurs differently.

During the moving process from $t_{1}$ to $t_{2}$, the relationship between the acceleration and the force is expressed as follows:

(2) $$\left\{ \begin{array}{l} F_{E}+F_{M}=M_{c}\cdot a_{1}\;\left(\mathrm{pre}\text{-}\mathrm{tension}\right)\\ F_{E}-F_{M}=M_{c}\cdot a_{2}\;\left(\mathrm{pre}\text{-}\mathrm{compression}\right) \end{array}\right.$$

where $F_{E}$ is the electrostatic force, $F_{M}$ is the elastic restoring force, and $M_{c}$ is the mass of the beam. The acceleration is $a_{1}$ when the beam is under pre-tension, and the acceleration is $a_{2}$ when the beam is under pre-compression.

When the beam moves from $t_{2}$ to $t_{3}$, the relationship between the acceleration and the force is as follows:

(3) $$\left\{ \begin{array}{l} F_{E}-F_{M}=M_{c}\cdot a_{1}\;\left(\mathrm{pre}\text{-}\mathrm{tension}\right)\\ F_{E}+F_{M}=M_{c}\cdot a_{2}\;\left(\mathrm{pre}\text{-}\mathrm{compression}\right) \end{array}\right.$$

#### 2.1.3. Dynamic Model

During the oscillation of the beam, the restoring force $F_{M}$ is not only a drive force but also a hinder force. Therefore, we set the coefficient λ to reflect the acting direction of the elastic restoring force, and the dynamical model of the beam is as follows:

(4) $$M_{e}\cdot \ddot{y}+C\cdot \dot{y}+\lambda \cdot K_{E}\cdot y=\mathrm{sgn}(\dot{y})\cdot F_{E}$$

where $\ddot{y}$ is the beam-oscillating acceleration, $\dot{y}$ is the oscillating velocity, *y* is the oscillating displacement, and $F_{E}$ is electrostatic force. $K_{E}$ represents the elastic stiffness of the microbeam, and its value will be affected by the constraint. The detailed analysis of $K_{E}$ can be seen in Appendix B. Function sgn($\dot{y}$) is used to change the acting direction of electric force and keeps it the same as the direction of motion. $M_{e}$ is the equivalent mass of the oscillating beam, and *C* is the equivalent damping coefficient. These parameters are calculated in Appendix C. The results of the dynamic model are verified in Section 3.4.

### 2.2. Experiment Setup

**Figure 3:**
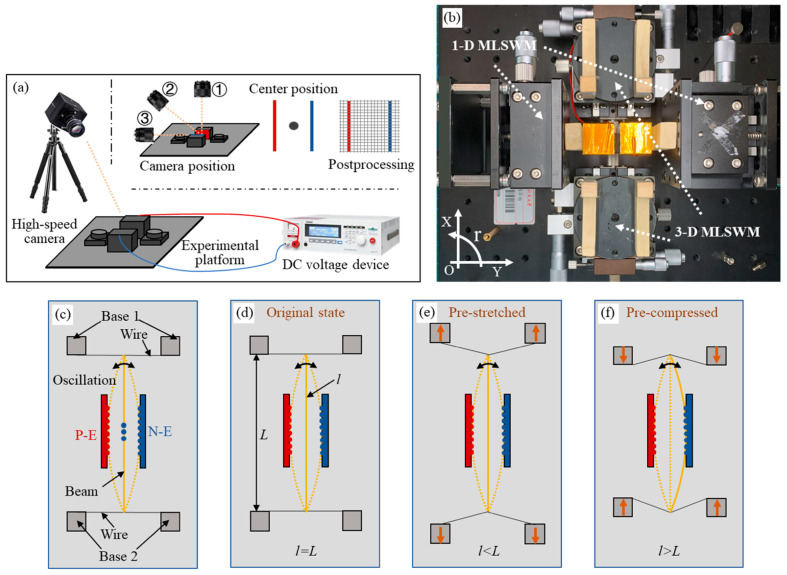
Experiment platform for the resonator under different pre-constraint conditions: (**a**) Schematics of the test system. (**b**) The photo (top view) of the experimental platform. (**c**) Installation position of the electrodes, beam, and constraint bases. (**d**) Schematics showing that when *l* = *L*, the microbeam is under the original constraint. (**e**) When *l* < *L*, the microbeam is under pre-stretched constraint. (**f**) When *l* > *L*, the microbeam is under pre-compressed constraint. Where the solid line means the stable position, the dotted line represents the oscillation process.

In Figure 3a, the test system is set up. The system consists of a manual mobile displacement platform, a high-speed camera (FASTCAM Mini UX 100 type 200K-C-32GB), and a DC power source (KIKUSUI Voltage Tester TOS9213AS). The camera has 3 positions for different observation goals. Position 1 is for capturing the overall oscillation of the beam from the top view, and Positions 2 and 3 are for capturing the tip motion of the beam from the side view. In the test, the camera must focus on the center point of the area between two electrodes. 

Figure 3b illustrates the photo of the experimental platform. The platform consists of two 1-D MLSWMs (one-dimensional manual linear stage with micrometer, SELN-LTP60-LM) that can move along Y direction and two 3-D MLSWMs (three-dimensional manual linear stage with micrometer, SELN-LX60-LMS) that can move along X, Y, and r direction. Two constraint bases of the resonator are, respectively, installed on two 3-D MLSWMs. In Figure 3c, the resonator consists of a microbeam, two constraint bases, and two plate electrodes. The microbeam is a copper foil with a length (*l*) of 40 mm, a thickness of 50 μm, and a width of 3 mm. Metal wires are utilized to support the beam. The metal wires are supported on two bases. Wood blocks are used to support the system to isolate the beam and experimental platform for test safety. The distance between the two electrodes is set as 4 mm. By changing the distance *L* of the two bases, we can subject the beam to different constraint conditions. When the beam length is equal to the distance of the two bases (*l* = *L*), the microbeam is under the original constraint, as shown in Figure 3d. When *l* < *L*, the microbeam is under pre-tension, as shown in Figure 3e. When *l* > *L*, the microbeam is under pre-compression, as shown in Figure 3f. For details of the microbeam-oscillation video when the beam is under different constraints, please refer to the Supplementary Materials in Video S1, Video S2, and Video S3.

### 2.3. Measurement Setup for Oscillating Velocity of the Microbeam

The working procedure of the experiment can be described as follows. The DC voltage is applied, and a steady electrostatic field is generated between the plate electrodes. Then, the microbeam oscillates under the influence of electrostatic force, elastic restoring force, and aerodynamic force. In this paper, the influence of variable elastic restoring force caused by different pre-constraints on the microbeam is tested. We adjust the 3-D MLSWMs to change the distance *L* between two constraint bases to subject the microbeam to the different pre-constraints. The coefficient $K_{L}$ is used to control the pre-constraint of the microbeam, which is determined as follows:

(5) $$K_{L}=\mathrm{sgn}(1-\frac{l}{L})(L-l)$$

where *l* is the beam length and *L* is the distance between constraint bases. When $K_{L}>0$, it represents that the beam is under pre-tension. When $K_{L}<0$, it means that the beam is under pre-compression.

During the oscillation, the motion track of the beam is recorded by the high-speed camera. After capturing the motion of the beam with a certain frame frequency *f*, the oscillating frequencies and instantaneous velocities of the beam during oscillation can be obtained. The detailed postprocessing results are shown in Section 3.3, and the raw experimental results are in the Supplementary Materials.

## 3. Results

### 3.1. Variable Axial Restrain Force $F_{N}$ Caused by Different Pre-Constraints

**Figure 4:**
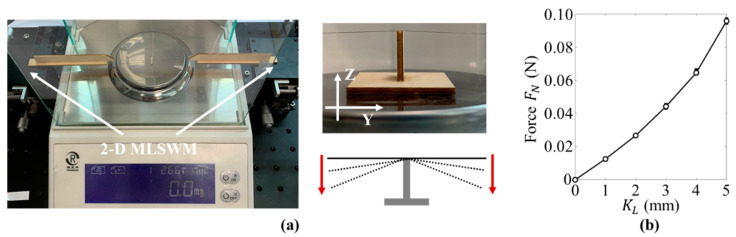
Force-displacement measurement for the anchor wire: (**a**) schematics of the test system; (**b**) the experimental results.

In the test, the displacement constraint is used to apply pre-constraint on the beam. Therefore, the influence of the value $K_{L}$ on the axial restrain force $F_{N}$ was studied first. We set up the simple experiment platform to measure the force $F_{N}$, as shown in Figure 4a. In the test, the anchor wire ends are assembled in the 2-D MLSWMs (they can move along the Y and Z directions), respectively. When we moved the platforms and changed the height of the wire from 0 mm to −3 mm at intervals of 0.5 mm (six conditions) and then changed the height back to 0 mm at the same intervals, the relationship between force $F_{N}$ and $K_{L}$ was obtained by using the electronic balance, as shown in Figure 4b. As $K_{L}$, increased, the force $F_{N}$ increased linearly, which supports our method of using the $K_{L}$ to describe the pre-constraint of the beam.

### 3.2. Variable Restoring Force $F_{M}$ Caused by Different Pre-Constraints $K_{L}$

**Figure 5:**
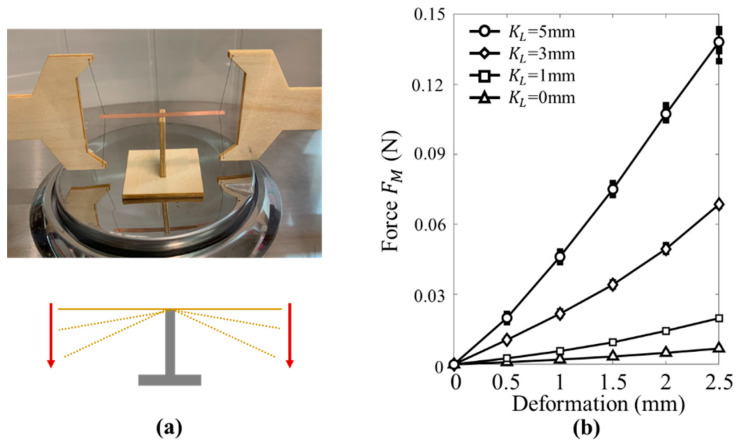
Experiment platform for the stiffness measurement of the beam: (**a**) schematics of the test system; (**b**) the experimental results.

As shown in the Figure 5a, the same measurement method applied in the Section 3.1 was used to study the stiffness of the beam. Additionally, the relationship between the quasi-steady restoring force $F_{M}$ and the deformation is obtained in Figure 5b. We can see that, as the beam deformation increased, the force $F_{M}$ increased linearly. In addition, the stiffness of the beam increased with the increase of the constraint coefficient $K_{L}$, which agrees well with the theoretical analysis of the beam in the Materials and Methods section. The larger $K_{L}$ means that the beam is under a larger axial restrain force $F_{N}$, and then the lateral deformation stiffness will be enhanced.

### 3.3. Variable Velocity-Position Characteristics Caused by Different Pre-Constraints

**Figure 6:**
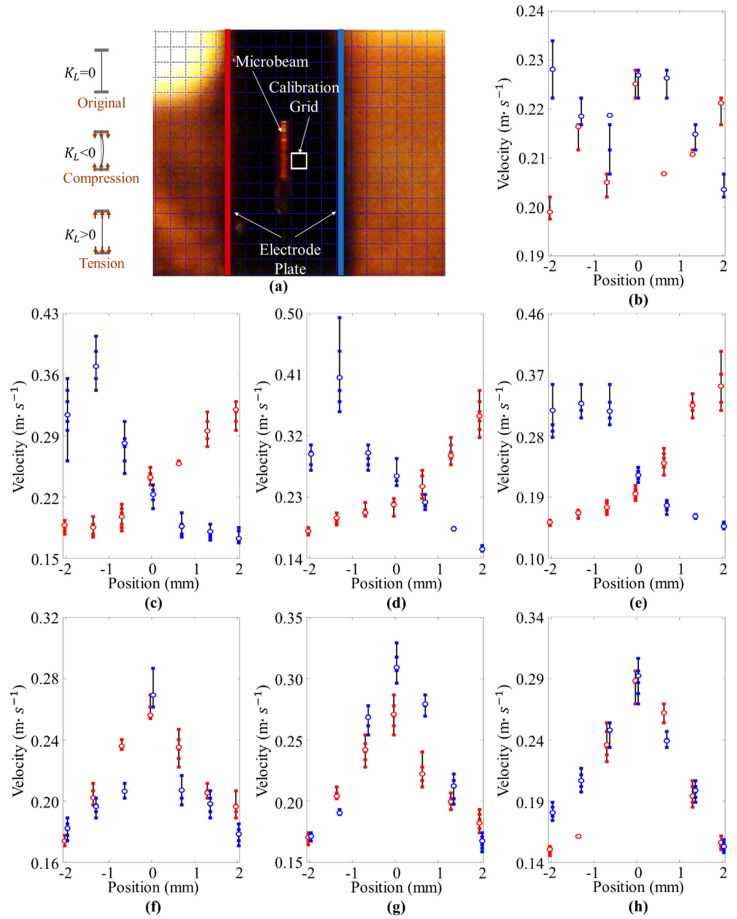
Experimental data of variable velocity-position characteristics under different constraint conditions: (**a**) snapshots of high-speed video; (**b**) the results under original constraint; (**c**–**e**) scatter-plot representation of the results under pre-compressed constraint, where (**c**) $K_{L}$ is −1 mm, (**d**) $K_{L}$ is −3 mm, and (**e**) $K_{L}$ is −5 mm; (**f**–**h**) scatter-plot representation of the results under pre-compressed constraint, where (**f**) $K_{L}$ is 1 mm, (**g**) $K_{L}$ is 3 mm, and (**h**) $K_{L}$ is 5 mm. The red points represent the result in the first half cycle, and the blue points represent the result in the second half cycle.

In the test, the value of $K_{L}$ is set from −5 mm to 5 mm at intervals of 2 mm, and the $K_{L}=0$ is also set as the standard condition (the whole test includes seven conditions). The positions −2 mm, 0 mm, and 2 mm are selected as three key observation positions. As shown in Figure 6a, after capturing the motion of the beam with a certain frame frequency *f*, the velocities of the beam passing through one calibration grid can be obtained. The oscillating velocity of the microbeam in one cycle is shown in Figure 6b–h. Besides, by recording the oscillating velocity of the beam in one cycle and taking the maximum velocity as the denominator, the velocity ratio (relative velocity) at different positions can be obtained as shown in Table 1.

In Figure 6, we can see that the beam can achieve variable velocity-position characteristics with the different $K_{L}$. When the beam is under pre-compression constraint, its oscillating velocity gets the maximum value around the position 2 mm and position −2 mm (the position near the electrodes), while when the beam is under pre-tension constraint, its oscillating velocity gets the maximum value around the position 0 mm (the middle position between two electrodes). Further, after observing the results of velocity ratio in Table 1, the maximum value is in the position −2 mm and 2 mm when $K_{L}<0$, and the maximum value is in the position 0 mm when $K_{L}>0$. In other words, the stable position of the beam is shifted, which is caused by the change in the action of elastic restoring force. This phenomenon is consistent with the previous theoretical analysis in Section 2.1.2.

### 3.4. Oscillation Simulation

**Figure 7:**
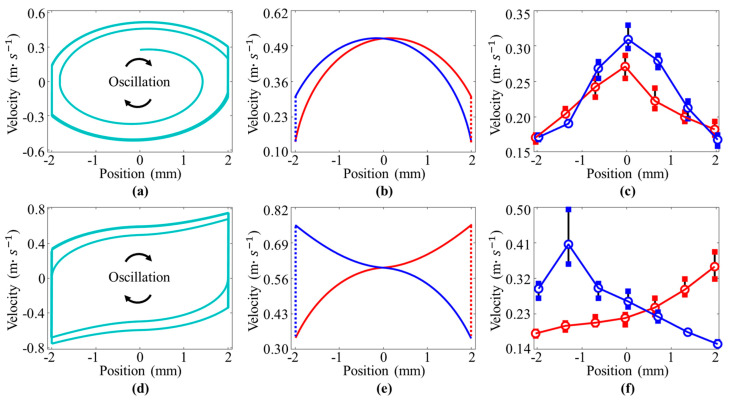
Simulation results of velocity-position curves: (**a**–**c**) experimental and numerical data for the pre-tension constraint; (**d**–**f**) experimental and numerical data for the pre-compression constraint. Velocity-position curves obtained by Equation (6) are shown in (**a**,**d**). Velocity-position curve in one cycle is depicted in (**b**,**e**). The experimental results in one oscillation cycle are pictured in (**c**,**f**). Where the red lines represent the data in the first half cycle, the blue lines represent the data in the second half cycle, ($K_{L}=\pm 3\;\mathrm{mm}$, and powering voltage V_DC_ = 2.5 KV).

We also conducted a simulation study for the resonator based on the dynamic model mentioned above. By solving Equation (4), the results of the velocity-position curves can be obtained under pre-tension constraint (as shown in Figure 7a) and pre-compression constraint (as shown in Figure 7d). Based on our dynamic model, we can also achieve the velocity output in one cycle. After comparing the numerical results shown in Figure 7b,e and the experiment results shown in Figure 7c,f, we can see that the simulation results agree well with the experimental results, but the numerical results are larger than the experiment results. The reasonable explanation for this phenomenon is as follows: when we installed the microbeam on the experimental platform, we supported it on the mobile platform through the anchor wire. There was friction between the wire and the mounting hole of the beam, but this damping force was not considered in the modeling process. Although there are certain errors between the model in this paper and the experimental results, the model reflects the variable velocity-position characteristics of the microbeam under different constraints. In addition, the theoretical model can also calculate the oscillating motion process of the double beam.

## 4. Discussion

### 4.1. Differential Motion of the Double-Beam-Resonator

**Figure 8:**
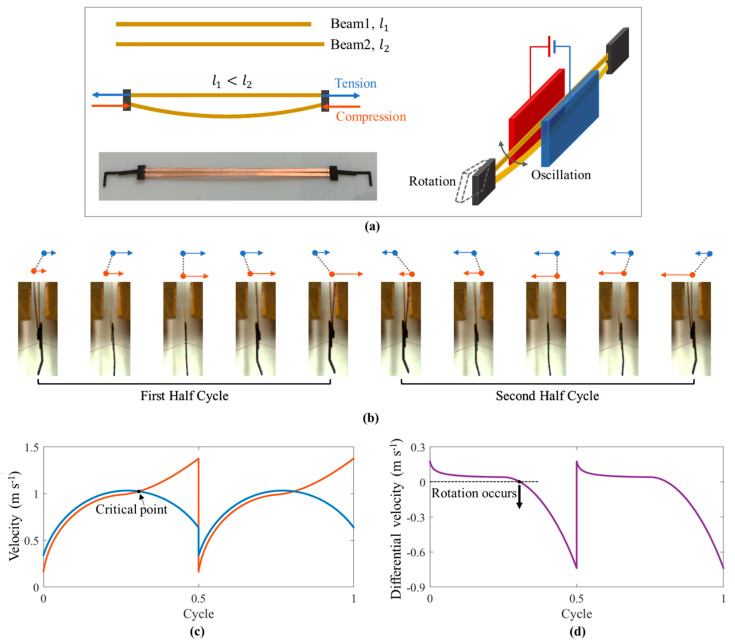
The differential motion of the two beams in the double-beam resonator: (**a**) the structure of the novel double-beam resonator; (**b**) the differential motion of the double-beam resonator is captured by the high-speed camera (the camera is posed in position 2 shown in Figure 2a); (**c**) the velocity output of each beam during one motion cycle; (**d**) the differential velocity output between two beams in one motion cycle, where the differential length of two beams is 1 mm, and the powering voltage is 4.5 KV.

According to the previous analysis of the single-beam resonator prototype, the beam demonstrated different velocity-position curves under different pre-constraints (pre-tension or pre-compression). Based on this feature, we present a double-beam resonator prototype to achieve differential motion of the beams, as shown in Figure 8a. The lengths of Beam 1 and Beam 2 are $l_{1}$ and $l_{2}$, respectively. When we connected the free ends of the two beams to form a double-beam structure, the short beam was in tension while the long beam was in compression. The novel double-beam structure can also oscillate between two electrodes under the DC voltage, while the two beams will oscillate with different velocity-position characteristics.

Based on the model of the single beam motion in Equation (4), the dynamic model of the double beam is as follows:

(6) $$M_{e}\cdot \ddot{Y}+C\cdot \dot{Y}+\lambda \cdot \mathrm{diag}\left(K_{E}\cdot Y\right)=\mathrm{sgn}\left(\dot{Y}\right)\cdot F_{E}$$

where the beam-oscillating acceleration is matrix $\ddot{Y}={\left[{\ddot{y}}_{1}\left(x,t\right),{\ddot{y}}_{2}\left(x,t\right)\right]}^{T}$, oscillating velocity is matrix $\dot{Y}={\left[{\dot{y}}_{1}\left(x,t\right),{\dot{y}}_{2}\left(x,t\right)\right]}^{T}$, oscillating displacement matrix is $Y=\left[y_{1},y_{2}\right]$, elastic stiffness is matrix $K_{E}={\left[K_{E1},K_{E2}\right]}^{T}$, and electrostatic force is matrix $F_{E}={\left[F_{E},F_{E}\right]}^{T}$. The subscripts 1 and 2 represent the parameters of Beam 1 and Beam 2, respectively. The function diag($K_{E}\dot{Y}$) is used to extract the main diagonal elements from the 2 × 2 matrix. Function sgn($\dot{Y}$) is used to change the acting direction of electric force and keeps it the same as the direction of motion. Other parameters can be obtained by the same method, as shown in Appendix C.

Figure 8b shows the photos of the motion for the double-beam resonator. We can see that the motions of the two beams are not synchronous; the differential motion of the two beams can be used to achieve the adjustment of the angle of attack during the oscillation. For example, at the beginning of Figure 8b, Beam 1 (blue points) moves faster than Beam 2 (red points); then, a positive angle of attack is created. Then, the double-beam structure keeps moving with a positive attack angle until Beam 2 moves faster than Beam 1. When the differential velocity of two beams achieves critical value, as shown in Figure 8c,d, the attack angle rotates from positive to negative. In a complete oscillation of the resonator, the attack angle is controlled by the differential motion of two beams to achieve an advanced rotation [2,21] with a very simple structure, which is attractive for the application of flapping-wing robotics.

### 4.2. The Properties of the Double-Beam Resonator Compared to Others

Table 2 shows the properties (velocity-position curve, frequency, active rotation) of the double-beam resonator compared to the single-beam resonator with different constraints. Compared with the single-beam that is simply supported, applying pre-tension constraint will increase the resonance frequency; applying pre-compression constraint will achieve different velocity-position curves. For the double-beam resonator, since the two beams are under the constraints of pre-compression and pre-tension, respectively, there will be a phase difference of velocity output to achieve advanced rotation of the whole structure.

### 4.3. Advanced Rotation Control of Flapping Wings

**Figure 9:**
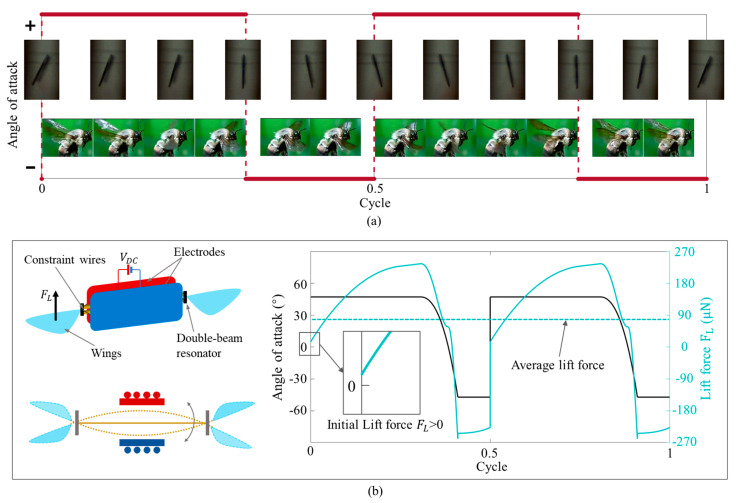
Advanced rotation control of flapping wings achieved by double-beam structure. (**a**) Photos of the advanced rotation of the double-beam structure compared with the bees’ wings. (**b**) Schematics of double-beam structure with flapping wings. Lift force and flapping attack angle of the double-beam resonator. The camera is posed in position 3, shown in Figure 4a.

From the analysis of the above sections, the differential motion of the double-beam resonator can achieve advanced rotation during its oscillation. In the article by Dickinson [2,3], an advance in rotation relative to translation will have a great influence on the lift force of flapping wings. Therefore, we added two wings on both sides of the double-beams structure and captured the wing motion using the high-speed camera, which is the same as the flapping process of bee wings in nature, shown in Figure 9a. After capturing the wing motions, we also calculated the lift force by using the theoretical model in reference [19]. The results are shown in Figure 9b. At the beginning of the motion cycle, the initial lift force generated by the wing is greater than 0, which is consistent with the advanced rotation characteristics in the literature [2,3].

It is noted that, although the applied DC voltage is relatively high, the required input power of the resonator is low due to the very small operating current. The high-voltage and small-current conditions, which can also be seen in the natural world, such as sparks created by removing a nylon shirt or discharges generated by electric eels, allow the resonator to be easily powered by many kinds of existing technologies.

## 5. Conclusions

By applying pre-tension or pre-compression constraints, a microbeam can achieve variable velocity-position characteristics under DC voltage, and the maximum velocity output position of the beam can be changed manually. Based on the single-beam structure, a double-beam resonator is also presented by utilizing the different velocity characteristics of the beam under different constraints. The novel double-beam resonator can achieve active rotation control of flapping wings with a very simple structure, and the microbeam can be made of a variety of conductive materials. All these features of our prototype are attractive in the field of microrobots.

## Figures and Tables

**Table 1:** The velocity ratio of the beam at different positions.

Cycle	First Half Cycle			Second Half Cycle		
Position/mm	−2	0	2	2	0	−2
$K_{L}=-5$ mm	44.06	56.02	100	42.31	63.84	90.14
$K_{L}=-3$ mm	45.34	55.04	87.16	38.62	65.31	73.44
$K_{L}=-1$ mm	50.95	65.94	86.74	46.90	60.50	84.32
$K_{L}=0$ mm	87.25	98.73	97.01	89.24	99.49	100
$K_{L}=1$ mm	64.78	95.11	73.07	66.33	100	67.83
$K_{L}=3$ mm	55.47	87.84	59.10	54.42	100	55.68
$K_{L}=5$ mm	51.86	99.03	53.78	52.67	100	62.07

**Table 2:** The Properties of the Different Resonator.

Resonator Prototype	Simply Supported 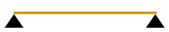	Pre-Tension 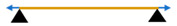	Pre-Compression 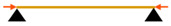	Double-Beam 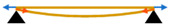
Velocity-Position Curve	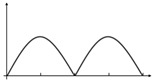	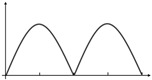	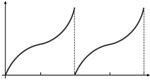	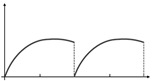
Frequency	Low	High	High	High
Active Rotation	No	No	No	Yes

**Figure A1:**
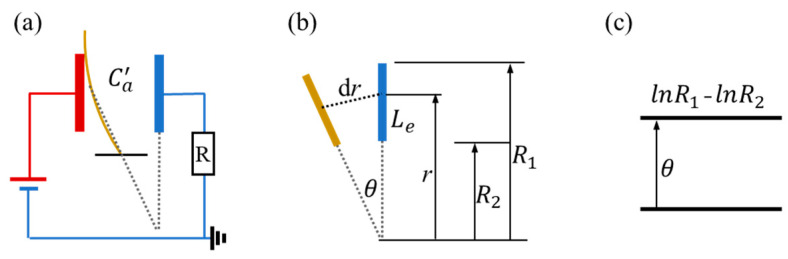
Schematic showing of the inclined plate-capacitor structure and its equivalent capacitance.

**Figure A2:**
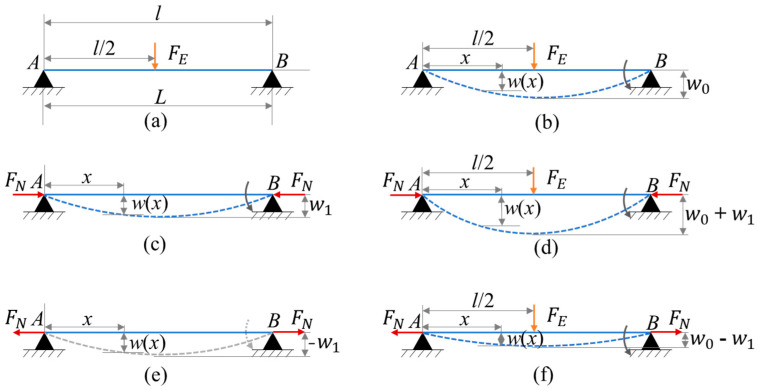
Schematic showing of the beam deformation under the action of external force $F_{E}$ and restraining force $F_{N}$: (**a**) the constraint of the beam; (**b**) deformation of the beam under the action of external force $F_{E}$; (**c**,**e**) deformation of the beam under the action of restraining force $F_{N}$; (**d**,**f**) deformation of the beam under the combined action of restraining force $F_{N}$ and external force $F_{E}$. The solid line represents the initial state, and the dotted line represents the deformation state.

**Figure A3:**
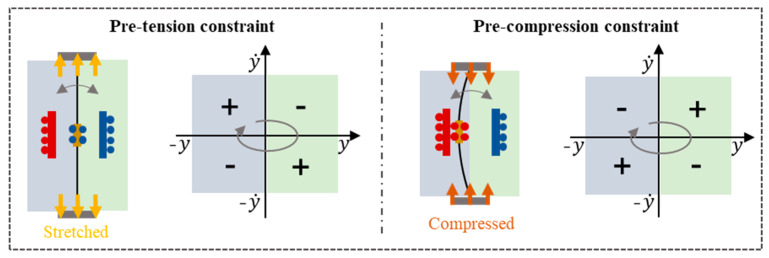
The value of λ when beam is under pre-tension or pre-compression constraint.

## Supplementary Materials

The following are available online at https://www.mdpi.com/article/10.3390/mi12060650/s1, PDF: Raw experimental results under Different Constraint Conditions, Videos S1–S4: Advanced rotation control of the resonator during the translation process.

## Appendix A. Calculation of Electrostatic Force

As shown in Figure A1, the inclined capacitor $C_{a}^{\prime }$ formed by the microbeam and electrode has an included angle θ. $L_{e}$ is the length of the electrode. $R_{2}$ and $R_{1}$ are the distances between the start and end points of the electrode, respectively. $w_{m}$ is the width of the micro-beam. $\varepsilon_{air}$ is the dielectric constant of air. For the non-parallel plate capacitors, conformal transformation can be used to convert them to the parallel plate capacitors. After the conversion, the electrode length is $\ln\left(R_{1}/R_{2}\right)$. The inclined capacitance is expressed as follows:

(A1) $$C_{a}^{\prime }=\frac{\varepsilon_{air}\varepsilon_{0}w_{m}}{\theta }\ln\frac{R_{1}}{R_{2}}\eta_{fringe}^{a}$$

Since the length and width of the electrodes are both limited, the edge effect needs to be considered in the calculation, where $\eta_{fringe}^{a}$ is the edge-effect factor [22,23] and the edge-effect factor $\eta_{fringe}^{a}$ is expressed as follows:

(A2) $$\eta_{fringe}^{a}=\left(1+\frac{d_{m}}{\pi l_{m}}\left[1+\ln\left(\frac{2\pi l_{m}}{d_{m}}\right)\right]\right)\left(1+\frac{d_{m}}{\pi w_{m}}\left[1+\ln\left(\frac{2\pi w_{m}}{d_{m}}\right)\right]\right)$$

$\Delta C_{a}$ is the part of capacitance formed by the positive and negative electrodes. Considering the edge effect, its value is as follows:

(A3) $$\Delta C_{a}=\frac{\varepsilon_{air}\varepsilon_{0}L_{e}w_{e}}{d}\left(1+\frac{d}{\pi L_{e}}\left[1+\ln\left(\frac{2\pi L_{e}}{d}\right)\right]\right)\left(1+\frac{d}{\pi w_{e}}\left[1+\ln\left(\frac{2\pi w_{e}}{d}\right)\right]\right)\frac{l_{m}w_{m}}{L_{e}w_{e}}$$

In this paper, the simulating parameters utilized for electrostatic force calculation are shown in Table A1.

**Table A1:** Parameter values of the simulation model.

$\mathrm{Parameters}\;\mathrm{Used}\;\mathrm{to}\;\mathrm{Calculate}\;F_{E}$	Value
Capacitance dielectric constant $\varepsilon_{0}$	8.86 × $10^{-12}$ F/m
Microbeam width $w_{m}$.	3 × $10^{-3}$ m
Electrode width $w_{e}$	20 × $10^{-3}$ m
Half distance between the electrodes $d_{m}$	2 × $10^{-3}$ m
Distance between the electrodes *d*	4 × $10^{-3}$ m
Electrode length $L_{e}$	20.4 × $10^{-3}$ m
Beam length between electrodes $l_{m}$	20 × $10^{-3}$ m
The angle between electrode and beam θ	0.1 ${\mathrm{rad}}^{-1}$
The top radius of the beam $R_{1}$	40.0667 × $10^{-3}$ m
The bottom radius of the beam $R_{2}$	20.0667 × $10^{-3}$ m

## Appendix B. Different Stiffness Caused by the Pre-Constraint

Assume that when only the force $F_{E}$ acts on the beam, the deflection is $w_{0}$, and when the beam length *l* > *L*, as shown in Figure A2c, the beam will be bent in the initial state with deflection $w_{1}$ under the action of axial restrain force $F_{N}$. In this condition, when the external force $F_{E}$ acts on the beam, it is regarded that an external force acts on the curved beam whose initial deflection is $w_{1}\left(x\right)$. As shown in Figure A2d, the deflection can be obtained by the following:

(A4) $$\frac{d^{2}w}{dx^{2}}-\frac{d^{2}w_{1}}{dx^{2}}=\frac{F_{E}x}{2EI}$$

When the beam oscillates upward, the initial deflection of the curved beam is $-w_{1}\left(x\right)$. It is regarded as through this process that the external force overcomes the deflection $w_{1}\left(x\right)$ to deform the beam. Under this condition, the deflection can be obtained by the following:

(A5) $$\frac{d^{2}w}{dx^{2}}+\frac{d^{2}w_{1}}{dx^{2}}=\frac{F_{E}x}{2EI}$$

where *E* is the elastic modulus of the material, *I* is the cross-sectional moment of inertia, and $w_{1}$ is the maximum deflection whose value is affected by the size of the coefficient $K_{L}$. The boundary conditions are w(0) = 0 and w(l) = 0. By solving Equations (A4) and (A5), the deflection of the beam is as follows:

(A6) $$\left\{ \begin{array}{l} w(x)=\frac{F_{E}x}{12EI}(x^{2}-l^{2})+w_{1}\sin(\frac{\pi }{l}x)\;\mathrm{downward}\\ w(x)=\frac{F_{E}x}{12EI}(x^{2}-l^{2})-w_{1}\sin(\frac{\pi }{l}x)\;\mathrm{upward} \end{array}\right.$$

When the beam length *l* < *L*, as shown in Figure A2e, the beam will be tensioned in a line in the initial state. Assume that the beam has a virtual deflection $-w_{1}\left(x\right)$ under the action of axial restrain force $F_{N}$. At this time, when the external force $F_{E}$ acts on the beam, the external force needs to overcome the virtual deflection $w_{1}\left(x\right)$ to deform the beam. As depicted in Figure A2f, the deflection can be obtained by the following formula:

(A7) $$\frac{d^{2}w}{dx^{2}}+\frac{d^{2}w_{1}}{dx^{2}}=\frac{F_{E}x}{2EI}$$

Under the same boundary conditions expressed previously, the deflection of the beam is as follows:

(A8) $$w(x)=\frac{F_{E}x}{12EI}(x^{2}-l^{2})+w_{1}\sin(\frac{\pi }{l}x)$$

During beam deformation, the equivalent elastic stiffness of the beam is obtained as follows:

(A9) $$K_{E}=\frac{F_{E}}{w(x)}$$

In this paper, the parameters in the elastic restoring force calculation are shown in Table A2.

**Table A2:** Structure parameters of the simulation model.

$\mathrm{Parameters}\;\mathrm{Used}\;\mathrm{to}\;\mathrm{Calculate}\;F_{M}$	Value
Microbeam width $w_{m}$.	3 × $10^{-3}$ m
Beam length l	40 × $10^{-3}$ m
Beam thickness $d_{th}$	50 × $10^{-6}$ m
Material modulus of elasticity E	11.9 × $10^{10}$ Pa
Cross-sectional moment of inertia I	1.5625 × $10^{-17}$ $m^{4}$

## Appendix C. Parameters for the Dynamic Model

The deflection of different parts along the X axis during motion can be expressed as follows:

(A10) $$y(x,t)=\frac{F_{E}x}{12EI}\left(\frac{3l^{2}}{4}-x^{2}\right)\cdot f(t)(x\leq \frac{l}{2})$$

The equivalent mass *M_e_* is as follows:

(A11) $$M_{e}=\frac{2\int_{0}^{\frac{l}{2}}\rho A{\dot{y}}^{2}(x,t)dx}{{\dot{y}}^{2}(\frac{l}{2},t)}$$

When the beam is oscillating, it is perpendicular to the direction of airflow, and the aerodynamic resistance per unit area of the beam is expressed as follows [19]:

(A12) $$dF_{air}=\frac{1}{2}C_{D}\rho_{air}{\dot{y}}^{2}(x,t)w_{m}dx$$

where $C_{D}$ is the aerodynamic damping coefficient, and $C_{D}$ is 3.4 [19], $\rho_{air}$ is the air density. The equivalent damping coefficient *C* is as follows:

(A13) $$C=\frac{C_{D}\rho_{air}\int_{0}^{\frac{l}{2}}{\dot{y}}^{2}(x,t)w_{m}dx}{{\dot{y}}^{2}(\frac{l}{2},t)}$$

λ is established to represent the resistance or drive action of elastic restoring force. Its expression is as follows:

(A14) $$\lambda =\mathrm{sgn}(K_{L})\cdot \mathrm{sgn}(\dot{y})\cdot \mathrm{sgn}(y)$$

where, in the same moment, the λ in the pre-tension condition is opposite to the λ in the pre-compression condition. Its value is shown in Figure A3.
