# Supplementary material for: An Electrostatic Self-Excited Resonator with Pre-Tension/Pre-Compression Constraint for Active Rotation Control
Source: Micromachines (Basel). 2021 Jun 1;12(6):650. doi: 10.3390/mi12060650 (PMC8227978; doi:10.3390/mi12060650)
Supplement: Supplementary file 1 [file micromachines-12-00650-s001.zip › Supplement material- Figures and formular.pdf]

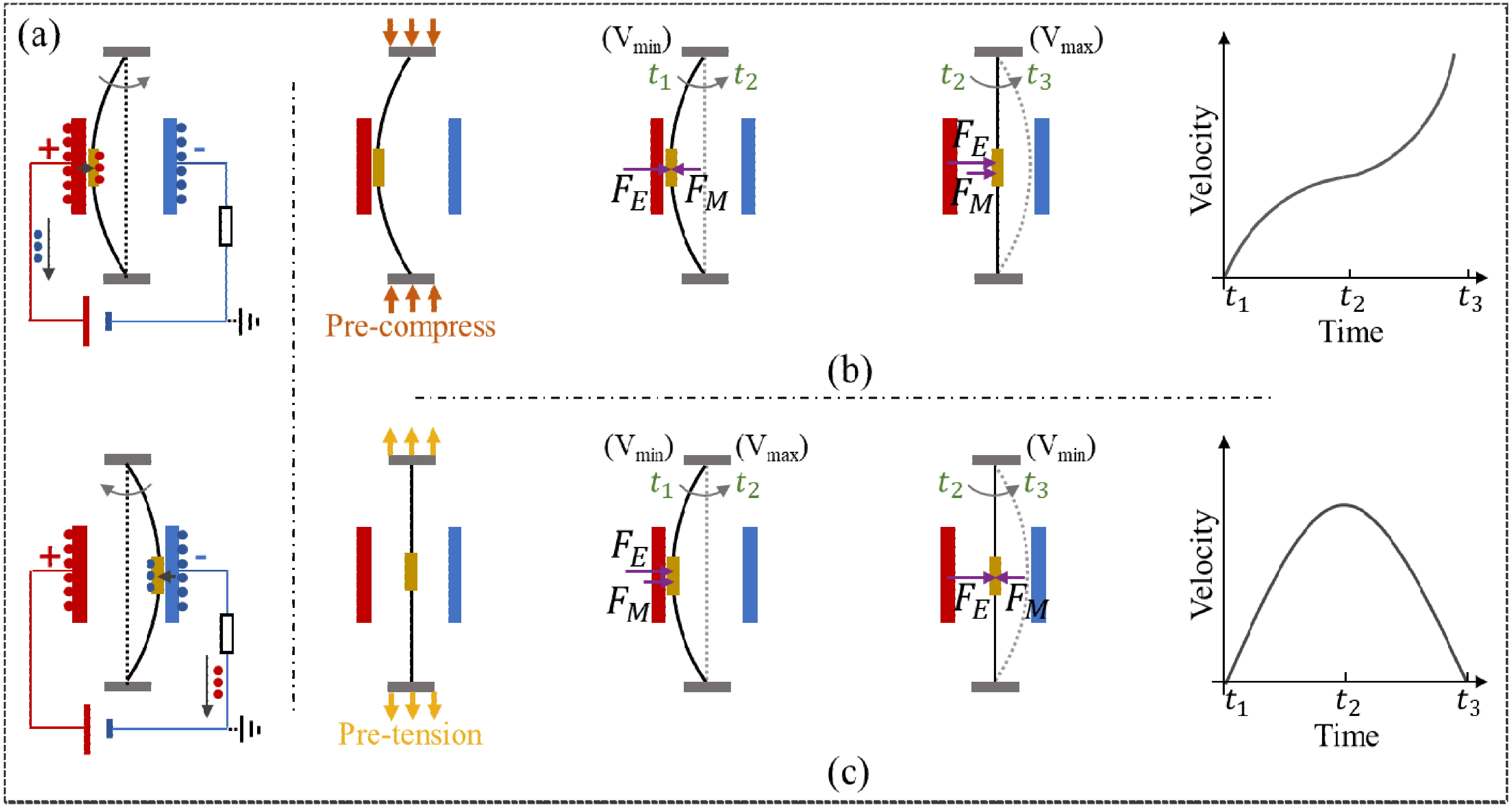

Figure S1. Structure, working principle, and force analysis of the electrostatic self-excited resonator with pre-compress and pre-tension constraints: (a) The charge flows in the circuit when the beam contacts with the positive and negative electrode. (b) The force analysis of the microbeam under pre-compression constraint. (c) The force analysis of the microbeam under pre-tension constrain.

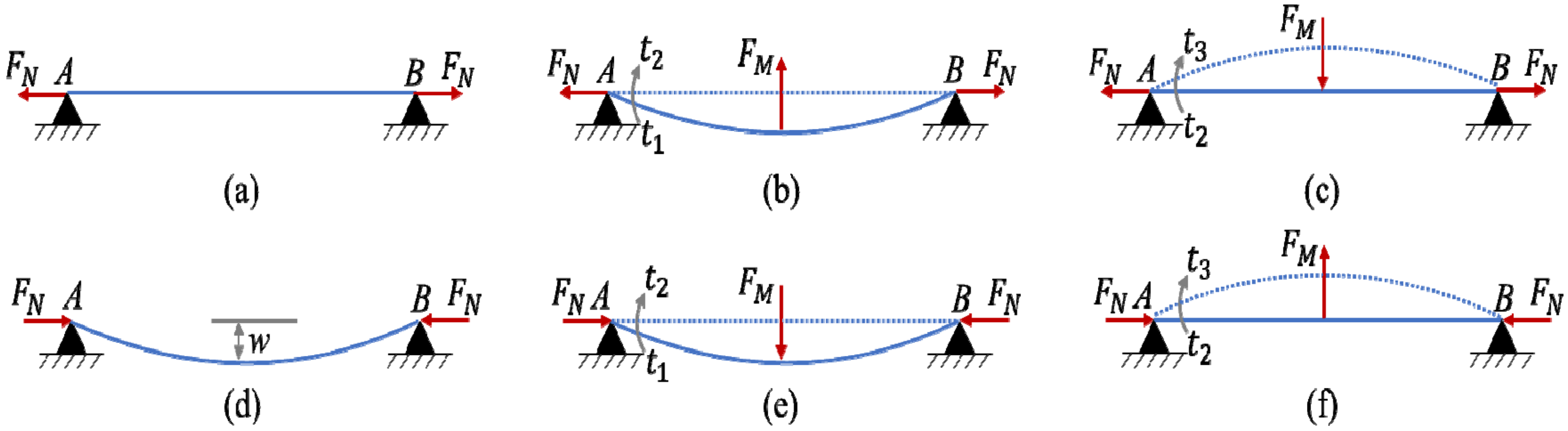

Figure S2. Quasi-steady force analysis of the self-excited resonator with pre-compress and pre-tension constraints for the shift of stable position. (a-c) Vibration process of the beam under pre-tension; (d-f) Vibration process of the beam under pre-compression. (a) and (d) are the stable position; (b) and (e) are the dynamic process from t_1 to t_2; (c) and (f) are the dynamic process from t_2 to t_3. The solid lines mean the position at current moment, and the dotted lines represent the position in the next step.

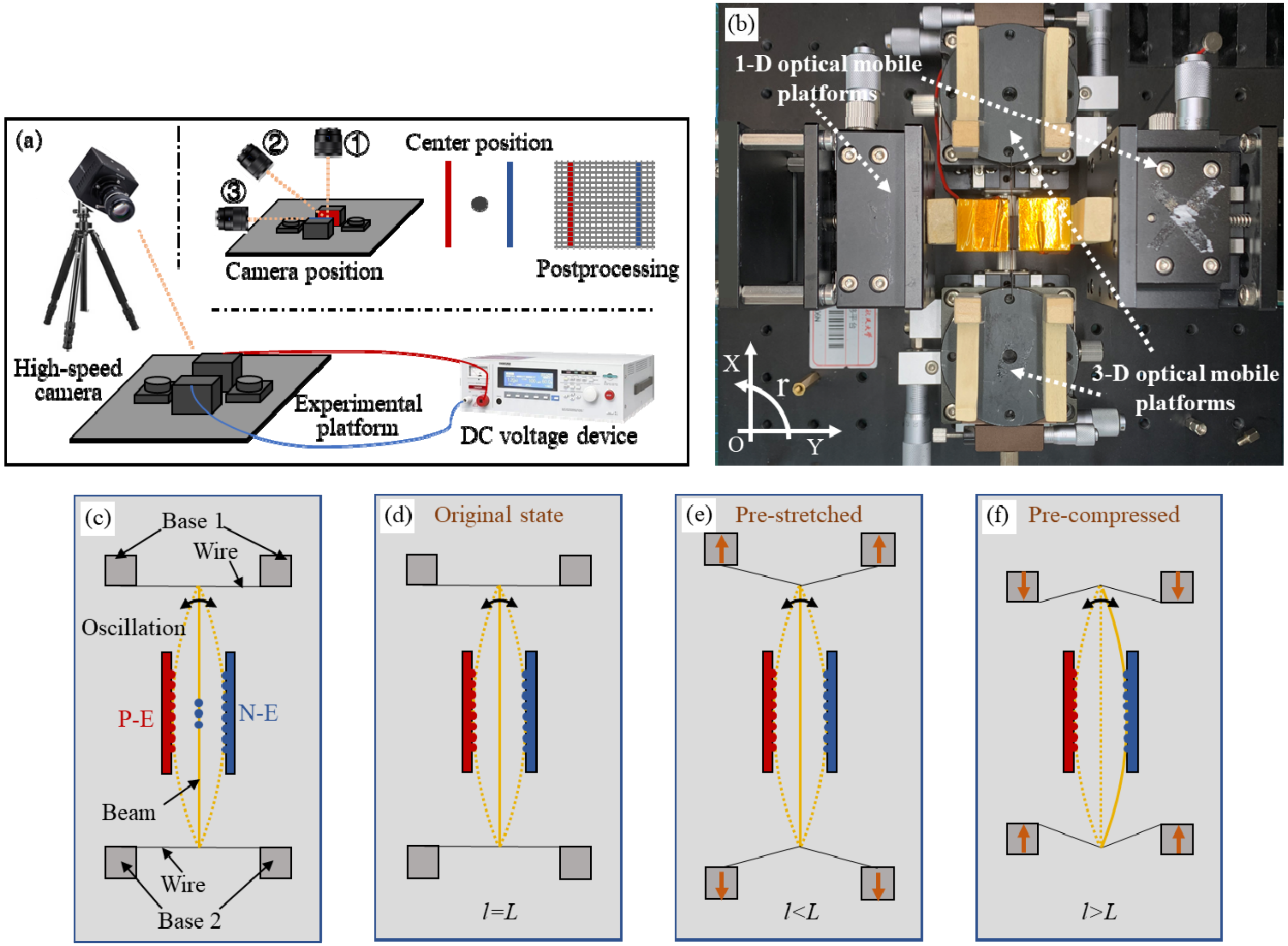

Figure S3. Schematics showing the experiment platform and the oscillating process under different pre-constraint conditions: (a) The test system using a high-speed camera to capture the microbeam motion. (b) Top view of the experimental platform. (c) Installation position of electrodes, beam and constraint bases. Where, solid line means the initial state and dotted line means oscillating process. (d) When $l=L$, micro-beam is under the original constraint. (e) When $l<L$, micro-beam is under the pre-stretched constraint. (f) When $l>L$, beam is under the pre-compressed constraint.

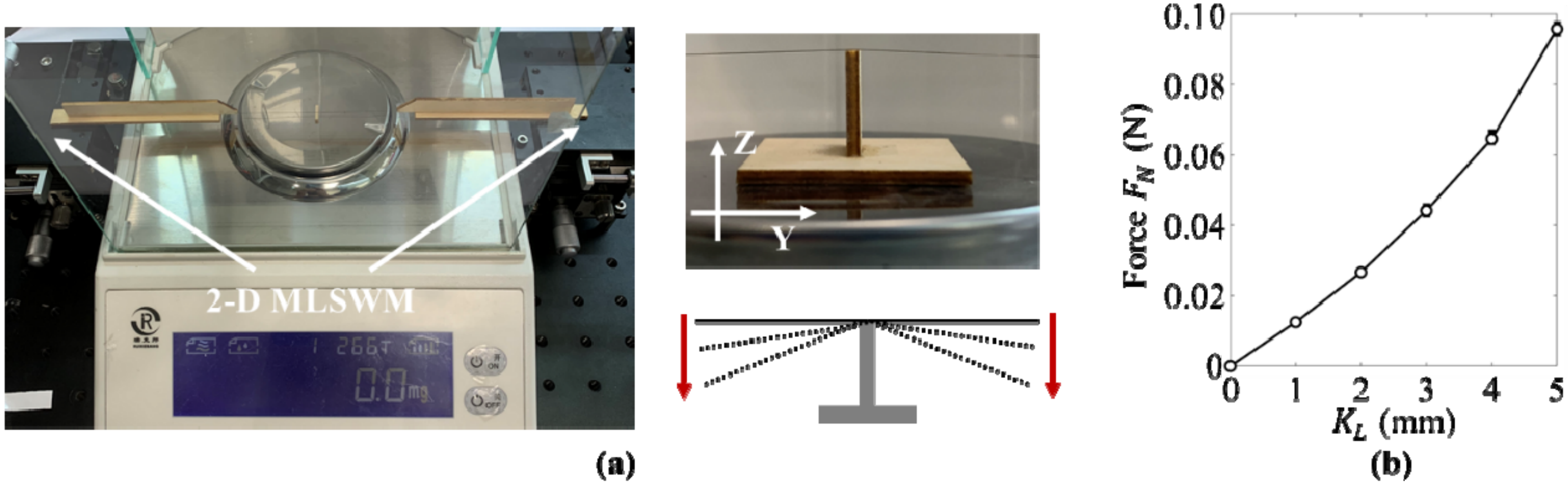

Figure S4. Force-displacement measurement for the anchor wire: (a) Schematics of the test system. (b) The experimental results.

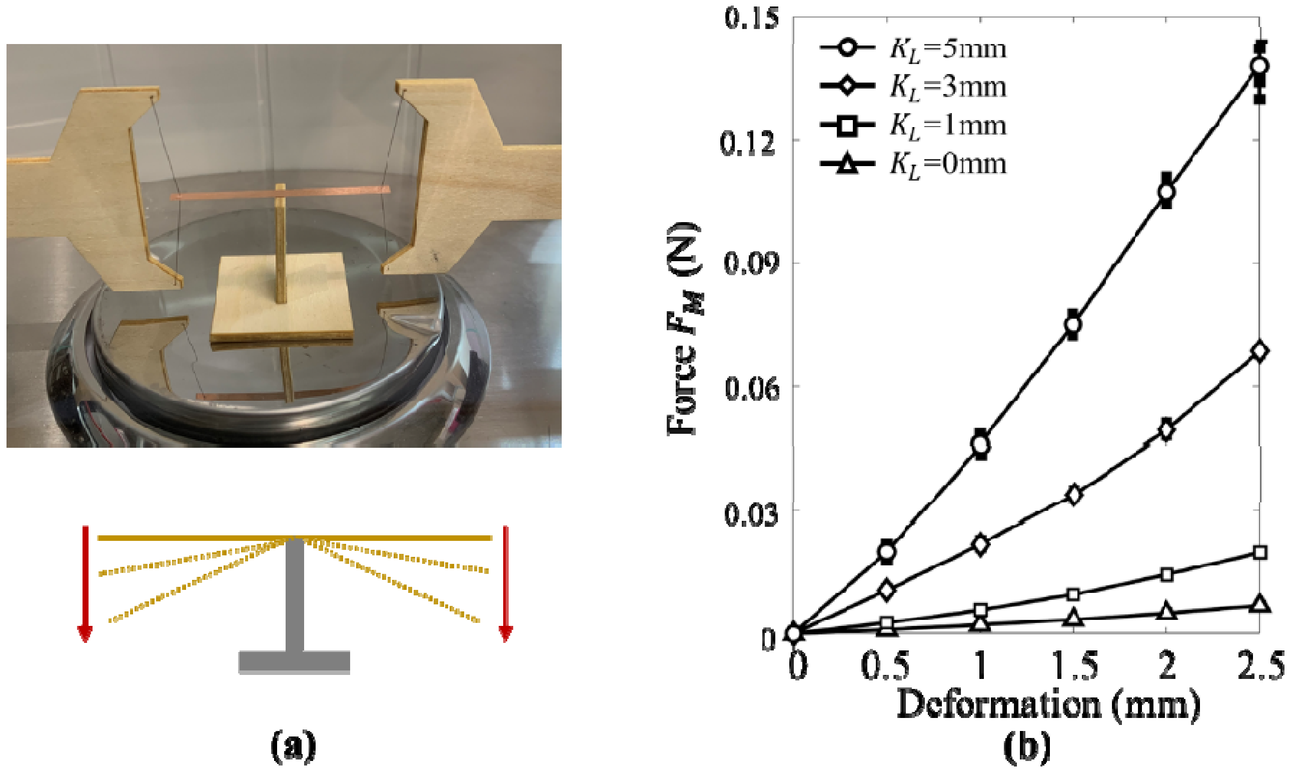

Figure S5. Experiment platform for the stiffness measurement of the beam: (a) Schematics of the test system. (b) The experimental results.

Figure S6. Experimental data of variable velocity-position characteristics under different constraint conditions. (a) snapshots of high-speed video. (b) the results under original constraint. (c-e) scatter-plot representation of the results under pre-compressed constraint, where (c) K_L is -1mm; (d) K_L is -3mm, and (e) K_L is -5mm. (f-h) scatter-plot representation of the results under pre-compressed constraint, where (f) K_L is 1mm, (g) K_L is 3mm, and (h) K_L is 5mm. The red points mean the result in the first half cycle and the blue points represent the result in the second half cycle.

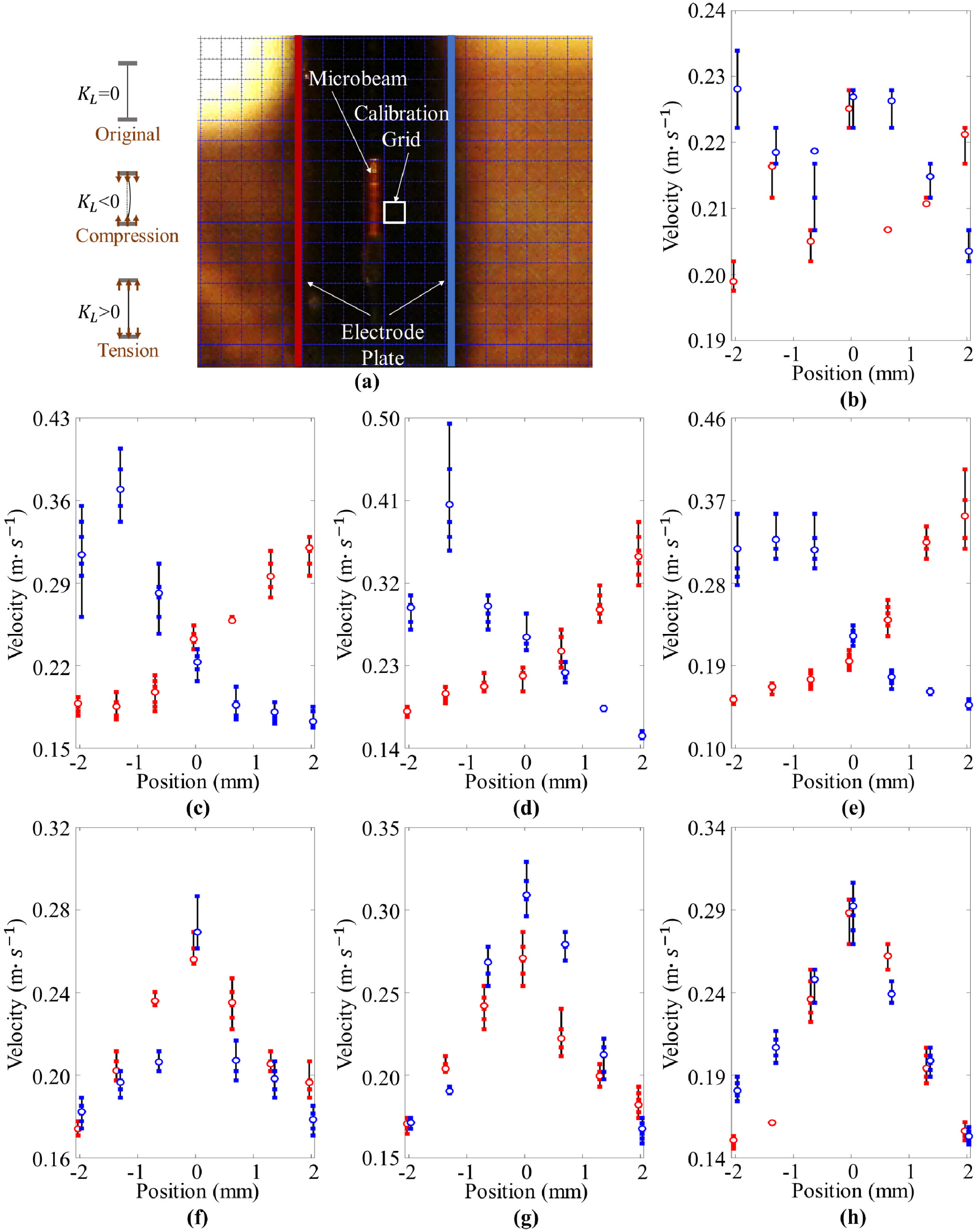

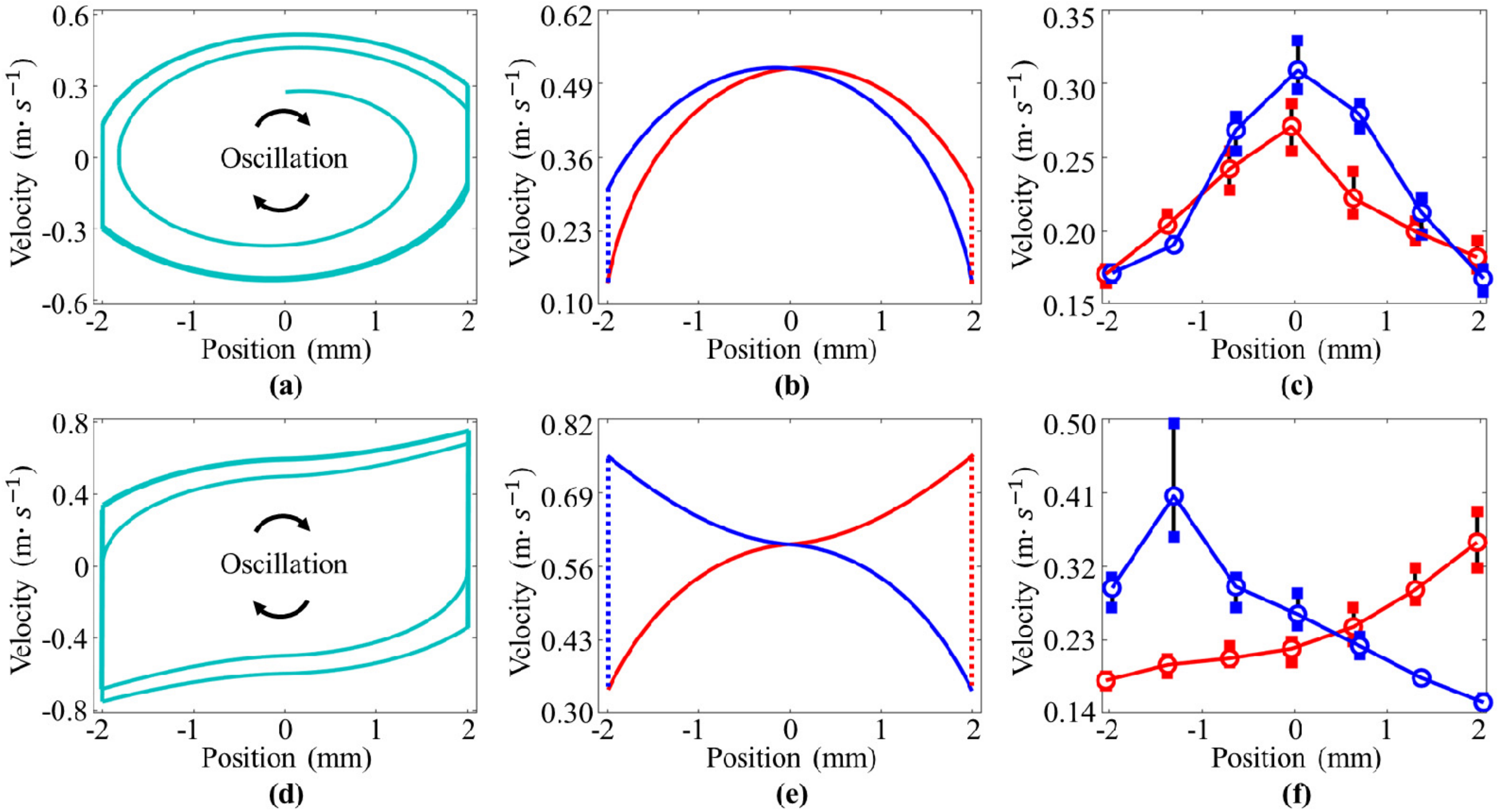

Figure S7. Simulation results of velocity-position curves: (a-c) Experimental and numerical data for the pre-tension constraint. (d-f) Experimental and numerical data for the pre-compression constraint. Velocity–position curves obtained by Eq. (6) are shown in Fig.(a) and (d). Velocity–position curve in one cycle is depicted in Fig.(b) and (e). The experimental results in one oscillation cycle are pictured in Fig.(c) and (f). Where, the red lines represent the data in the first half cycle, the blue lines mean the data in the second half cycle, ($K_L = \pm 3$mm, and powering voltage V_{DC}=2.5KV).

Figure S8. The differential motion of the two beams in the double-beam resonator. (a) The structure of the novel double-beam resonator. (b) The differential motion of the double-beam resonator is captured by the high-speed camera (The camera is posed in position 2 shown in Fig.2(a)). (c) The velocity output of each beam during one motion cycle. (d) The differential velocity output between two beams in one motion cycle. Where the differential length of two beams is 1mm, and the powering voltage is 4.5KV.

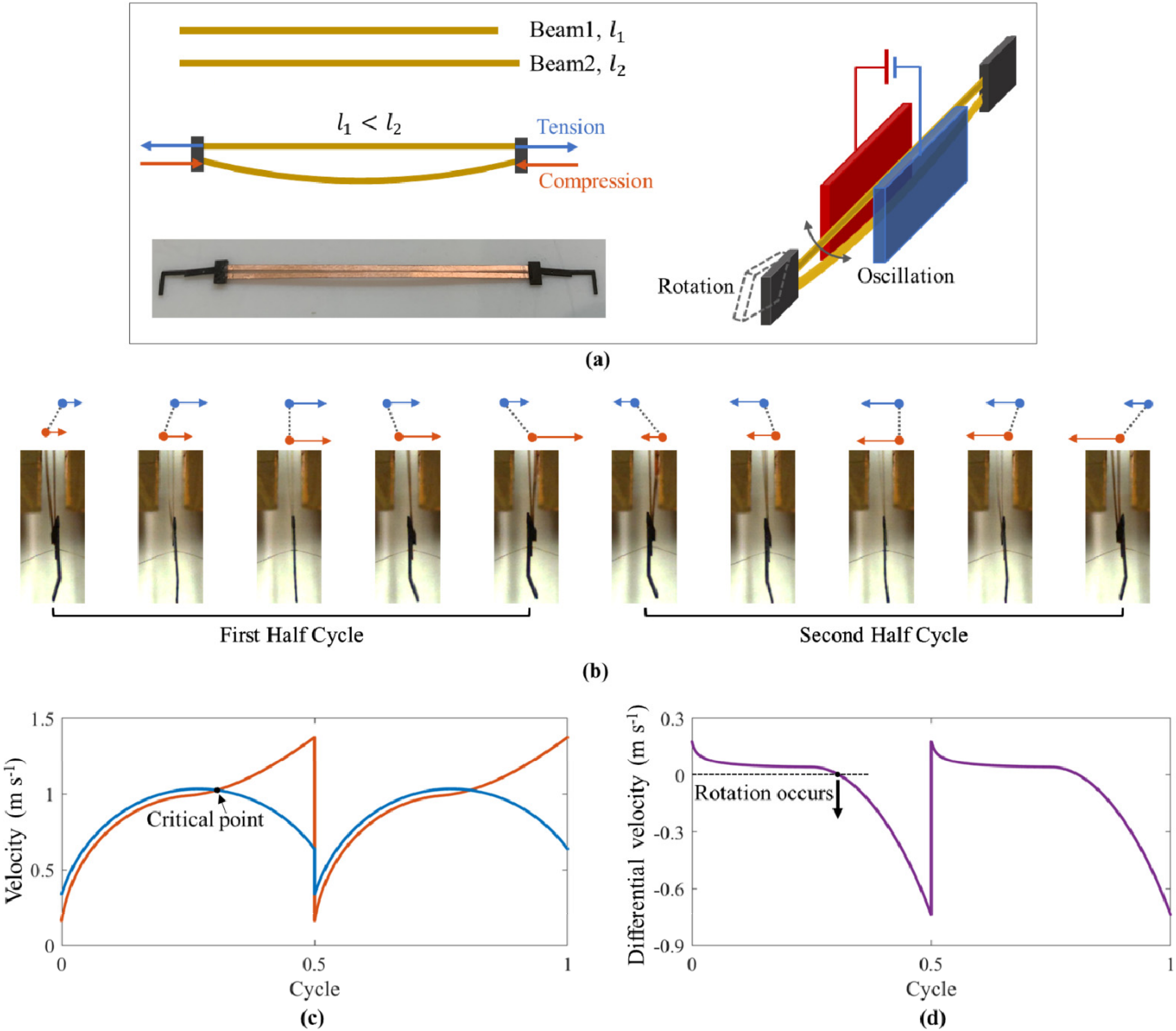

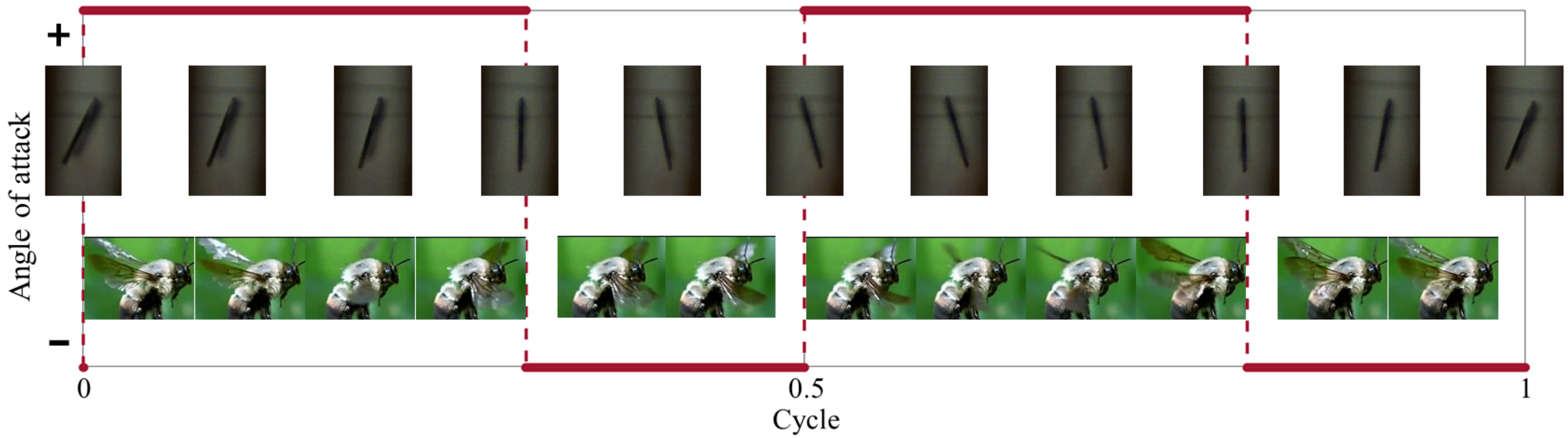

(a)

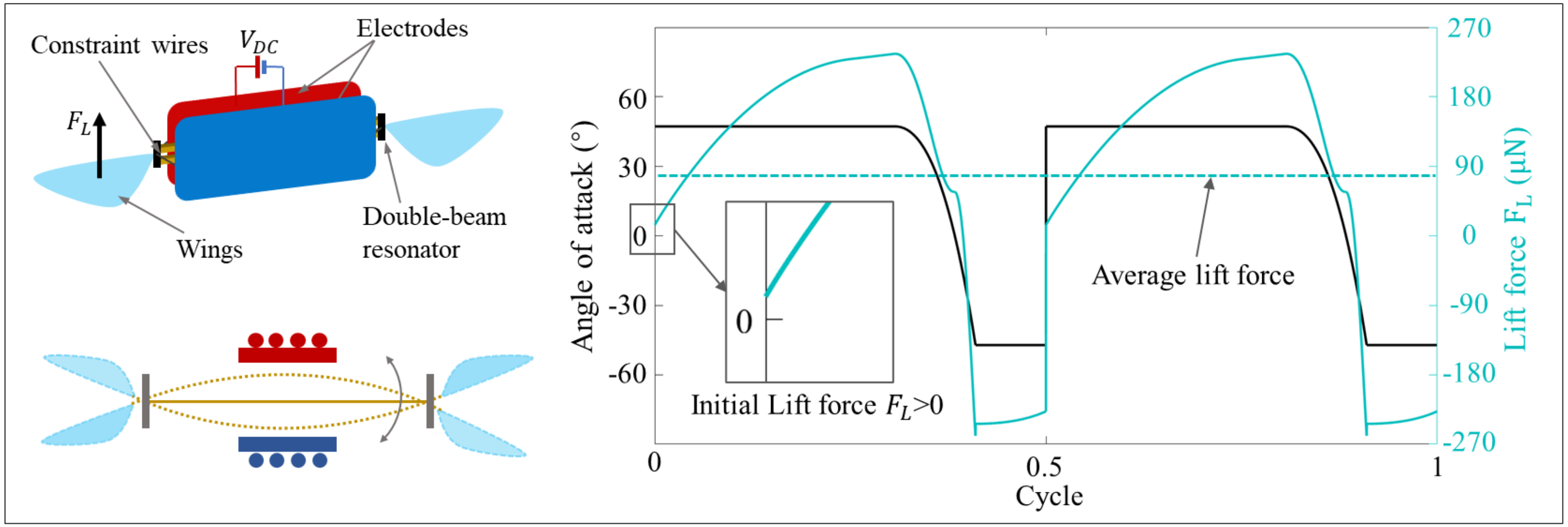

(b)

Figure S9. Advanced rotation control of flapping wings achieved by double-beam structure. (a) Photos of the advanced rotation of the double-beam structure compared with the bees' wings. (b) Schematics of double-beam structure with flapping wings. Lift force and flapping attack angle of the double-beam resonator. Where, the camera is posed in position 3 shown in Fig.4(a).

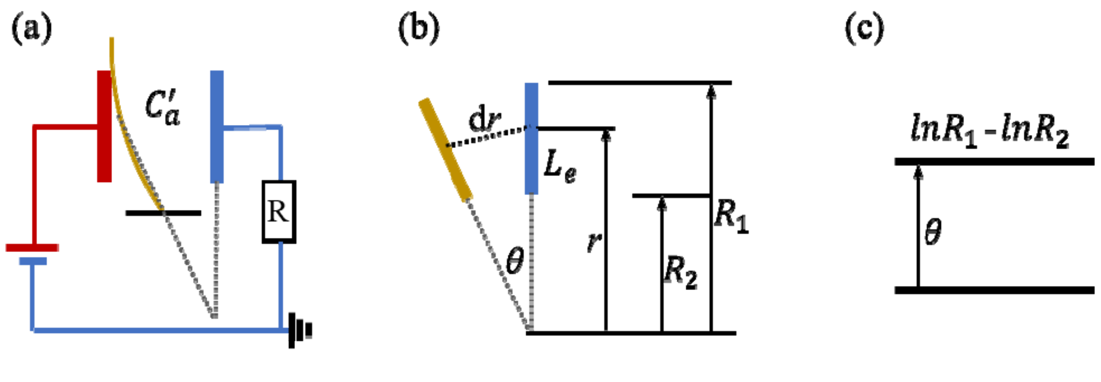

Figure A1. Schematic showing of the inclined plate capacitor structure and its equivalent capacitance.

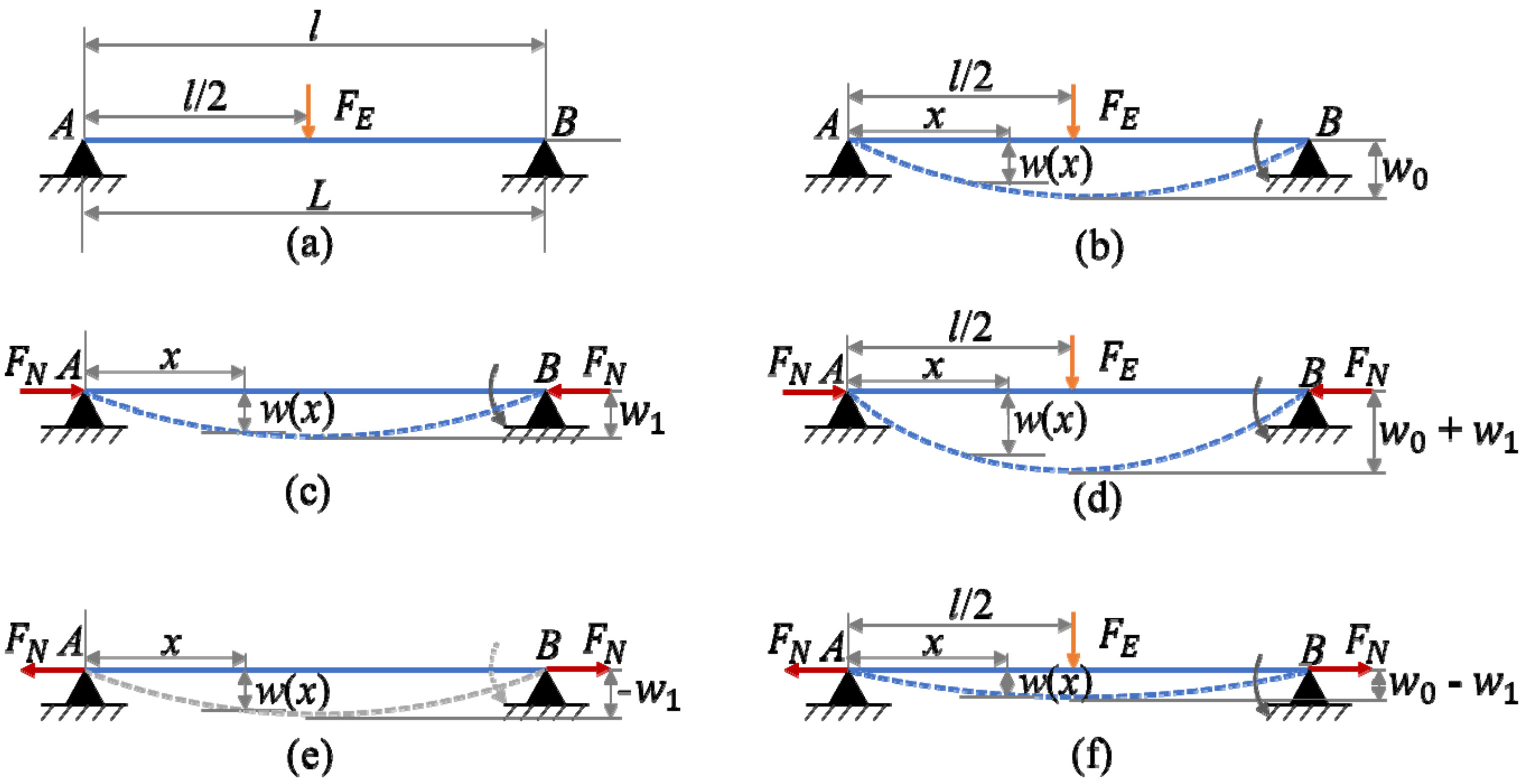

Figure A2. Schematic showing of the beam deformation under the action of external force F_E and restraining force F_N: (a) The constraint of the beam. (b) Deformation of beam under the action of external force F_E. (c, e) Deformation of beam under the action of restraining force F_N. (d, f) Deformation of beam under the combined action of restraining force F_N and external force F_E. Solid line represents initial state and dotted line represents deformation state.

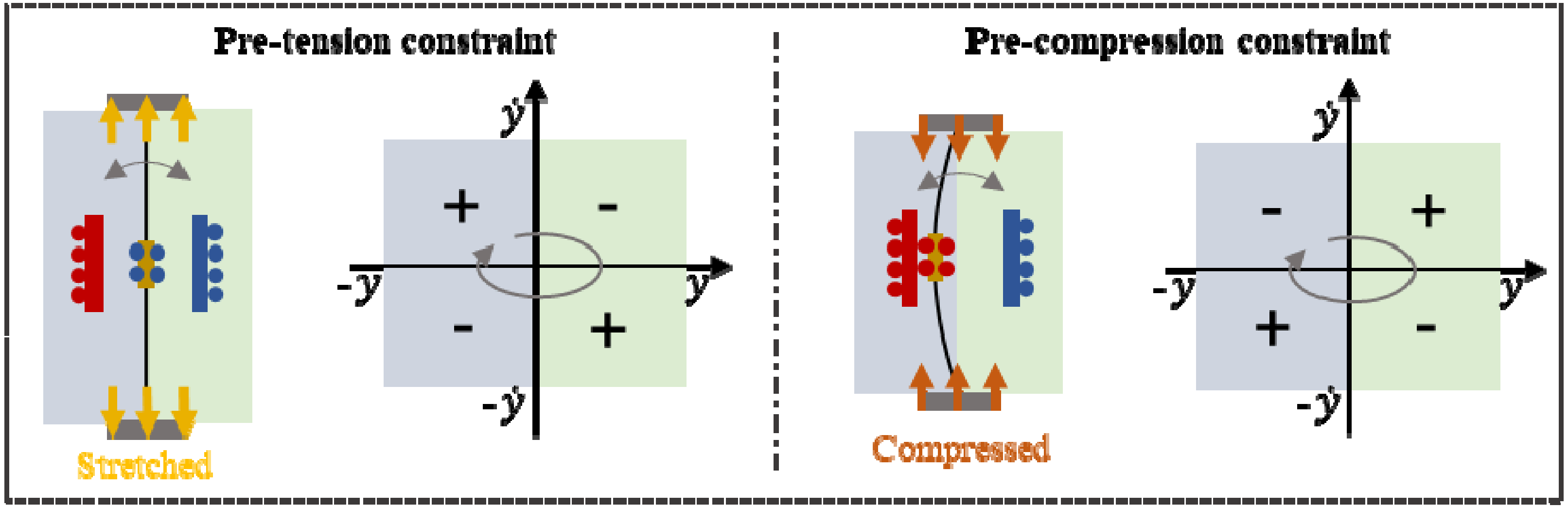

Figure A3. The value of λ when beam is under pre-tension or pre-compression constraint.

Formular: 1-6

$$F_E = \left(C_a' - \Delta C_a\right)\frac{U^2}{d}$$

$$\begin{cases} F_E + F_M = M_c \cdot a_1 & \text{(pre-tension)} \\ F_E - F_M = M_c \cdot a_2 & \text{(pre-compression)} \end{cases}$$

$$\begin{cases} F_E - F_M = M_c \cdot a_1 & \text{(pre-tension)} \\ F_E + F_M = M_c \cdot a_2 & \text{(pre-compression)} \end{cases}$$

$$M_e \cdot \ddot{y} + C \cdot \dot{y} + \lambda \cdot K_E \cdot y = \operatorname{sgn}(\dot{y}) \cdot F_E$$

$$K_L = \operatorname{sgn}(1 - \frac{l}{L})(L - l)$$

$$M_e \cdot \ddot{\mathbf{Y}} + C \cdot \dot{\mathbf{Y}} + \lambda \cdot \operatorname{diag}\left(\mathbf{K_E} \cdot \mathbf{Y}\right) = \operatorname{sgn}\left(\dot{\mathbf{Y}}\right) \cdot \mathbf{F_E}$$

Formular: A.1-A.3

$$C_a' = \frac{\varepsilon_{air}\varepsilon_0 w_m}{\theta} \ln\frac{R_1}{R_2}\eta_{fringe}^{a}$$

$$\eta_{fringe}^{a} = \left(1+\frac{d_m}{\pi l_m}\left[1+\ln\left(\frac{2\pi l_m}{d_m}\right)\right]\right)\left(1+\frac{d_m}{\pi w_m}\left[1+\ln\left(\frac{2\pi w_m}{d_m}\right)\right]\right)$$

$$\Delta C_a = \frac{\varepsilon_{air}\varepsilon_0 L_e w_e}{d}\left(1+\frac{d}{\pi L_e}\left[1+\ln\left(\frac{2\pi L_e}{d}\right)\right]\right)\left(1+\frac{d}{\pi w_e}\left[1+\ln\left(\frac{2\pi w_e}{d}\right)\right]\right)\frac{l_m w_m}{L_e w_e}$$

Formular: B.1-B.6

$$\frac{\mathrm{d}^2 w}{\mathrm{d}x^2} - \frac{\mathrm{d}^2 w_1}{\mathrm{d}x^2} = \frac{F_E x}{2EI}$$

$$\frac{\mathrm{d}^2 w}{\mathrm{d}x^2} + \frac{\mathrm{d}^2 w_1}{\mathrm{d}x^2} = \frac{F_E x}{2EI}$$

$$\begin{cases} w(x) = \dfrac{F_E x}{12EI}(x^2 - l^2) + w_1 \sin(\dfrac{\pi}{l} x) & \text{downward} \\ w(x) = \dfrac{F_E x}{12EI}(x^2 - l^2) - w_1 \sin(\dfrac{\pi}{l} x) & \text{upward} \end{cases}$$

$$\frac{\mathrm{d}^2 w}{\mathrm{d}x^2} + \frac{\mathrm{d}^2 w_1}{\mathrm{d}x^2} = \frac{F_E x}{2EI}$$

$$w(x) = \frac{F_E x}{12EI}(x^2 - l^2) - w_1 \sin(\frac{\pi}{l} x)$$

$$K_E = \frac{F_E}{w(x)}$$

Formular: C.1-C.5

$$y(x,t) = \frac{F_E x}{12EI}\left(\frac{3l^2}{4} - x^2\right)\cdot f(t) \quad (x \le \frac{l}{2})$$

$$M_e = \frac{2\int_0^{\frac{l}{2}} \rho A \dot{y}^2(x,t)dx}{\dot{y}^2(\frac{l}{2},t)}$$

$$dF_{air} = \frac{1}{2} C_D \rho_{air} \dot{y}^2(x,t) w_m dx$$

$$C = \frac{C_D \rho_{air} \int_0^{\frac{l}{2}} \dot{y}^2(x,t) w_m dx}{\dot{y}^2(\frac{l}{2},t)}$$

$$\lambda = \mathrm{sgn}(K_L)\cdot \mathrm{sgn}(\dot{y})\cdot \mathrm{sgn}(y)$$
