# Supplementary material for: An Electrostatic Self-Excited Resonator with Pre-Tension/Pre-Compression Constraint for Active Rotation Control
Source: Micromachines (Basel). 2021 Jun 1;12(6):650. doi: 10.3390/mi12060650 (PMC8227978; doi:10.3390/mi12060650)
Supplement: Supplementary file 1 [file micromachines-12-00650-s001.zip › Supplementary material-Raw experimental results.pdf]

							Recorded data				
**Observation | K_L=-5mm**	**mean value**	**1**	**2**	**3**	**4**	**5**	**6**	**7**	**8**	**9**	**10**
**1**	5.8	5.8	5.7	5.8	5.8	5.6	5.7	5.7	5.9	6	6.1
**2**	5.3	5.2	5.4	5.1	5.1	5.3	5.7	5.2	5.4	5.3	5.6
**3**	5.1	4.8	5.1	5.2	5.3	5.4	5.1	4.7	4.9	4.8	5.6
**4**	4.6	4.8	4.6	4.5	4.9	4.3	4.7	4.7	4.4	4.6	4.2
**5**	3.7	3.5	3.8	4	4.4	3.6	3.8	3.8	3.3	3.7	3.4
**6**	2.8	2.9	2.5	2.7	2.6	2.8	2.5	2.8	2.9	3.1	2.7
**7**	2.6	3.2	2.7	2.8	2.2	2.4	2.4	2.8	2.4	2.7	2
**1**	6.1	6	5.9	5.7	6.2	6.1	6.2	5.8	6.4	6.2	6
**2**	5.5	5.6	5.5	5.5	5.3	5.8	5.5	5.3	5.8	5.4	5.3
**3**	5	4.9	4.8	4.7	5.6	5.4	5.1	4.7	4.8	5	5.2
**4**	4	3.7	4.1	3.8	3.9	4.1	4.3	3.7	4.3	4.2	4
**5**	2.9	2.7	2.9	3	3	2.8	2.8	3.4	2.3	2.5	3
**6**	2.7	2.8	3	3	2.5	2.8	2.5	2.4	2.9	2.7	2.7
**7**	2.8	3	2.5	3.3	3.2	3	3	2.4	3.1	2.5	2.4
**Total number of frames in 30 cycles**						1814					

							Recorded data				
**Observation | K_L=-3mm**	**mean value**	**1**	**2**	**3**	**4**	**5**	**6**	**7**	**8**	**9**	**10**
**1**	4.9	4.6	4.8	4.9	5.1	4.8	5.3	4.8	5	5.1	5
**2**	4.5	4.7	4.6	4.3	4	4.9	4.3	4.5	4.6	4.5	4.3
**3**	4.3	4.4	4.4	3.9	4	4.2	4.3	4.6	4.2	4.6	4.4
**4**	4.1	3.9	4	4	4	4.1	4	4.5	4	4.4	3.8
**5**	3.6	3.8	3.6	3.9	3.8	3.1	3.4	3.3	3.8	4.1	3.6
**6**	3.1	3.2	3.1	2.7	3.1	2.8	3.4	3	3.4	2.9	3.1
**7**	2.6	2.4	3.1	2.3	2.6	2.2	2.4	2.5	2.7	2.8	2.7
**1**	5.8	5.7	6	6	5.8	5.9	5.9	5.5	6	5.6	5.6
**2**	4.9	4.8	4.7	4.8	4.9	5.1	4.8	4.9	4.7	4.9	4.9
**3**	4	3.8	4.1	4.2	4	4.4	3.8	3.8	3.6	4.2	4.2
**4**	3.4	3.4	3.6	3.4	3.5	3.4	3.5	3.4	4.1	3.1	2.9
**5**	3	2.7	2.7	2.9	3	2.9	3.1	3.1	3.2	3.4	3.3
**6**	2.2	2	1.7	2.5	1.8	2.3	2.3	2.9	2.3	2.2	2.4
**7**	3.1	3.2	3.4	2.9	3.3	3.2	3	3.2	2.7	2.7	2.9
**Total number of frames in 30 cycles**						1693					

							Recorded data				
**Observation | K_L=-1mm**	**mean value**	**1**	**2**	**3**	**4**	**5**	**6**	**7**	**8**	**9**	**10**
**1**	4.8	4.9	5.2	4.8	4.9	4.6	4.6	4	4.8	5	4.7
**2**	4.8	5.3	4.8	5.1	4.7	4.8	5	5	4.5	4.4	4.5
**3**	4.5	4.9	5	4.5	4.3	4.8	4.4	4.4	4.7	4.2	4
**4**	3.7	3.7	3.7	3.6	3.7	3.9	3.8	3.4	3.8	3.6	3.5
**5**	3.5	3.6	3.1	3.4	3.4	3.6	3.6	3.6	3.4	3.4	3.4
**6**	3	3.3	2.6	3	3.2	2.9	3.2	3.1	2.8	3.1	3
**7**	2.8	2.8	2.7	2.9	2.4	2.8	2.8	2.7	3.1	2.7	3
**1**	5.2	5.4	5.2	5.3	5.4	5.2	5.3	4.8	5.4	4.9	4.7
**2**	4.9	5.3	5	5.1	4.9	4.7	4.2	5.2	4.9	5	5.1
**3**	4.8	4.7	4.7	5.1	5	4.8	4.4	4.4	4.3	5.5	5
**4**	4	3.9	4.5	4	4.1	3.7	3.8	3.7	4.1	4.3	3.9
**5**	3.2	2.9	3.9	3.4	2.9	2.8	3.2	3.6	3.1	3.2	2.9
**6**	2.4	2.8	2.4	2.5	2.2	2.6	2.5	2.1	2.4	2.3	2.4
**7**	2.9	2.5	3.4	3	2.7	3.5	3	2.9	2.7	2.6	2.4
**Total number of frames in 30 cycles**						1710					

							Recorded data				
**Observation | K_L=0**	**mean value**	**1**	**2**	**3**	**4**	**5**	**6**	**7**	**8**	**9**	**10**
**1**	4.5	4.3	4.5	4.6	4.5	4.3	4.4	4.5	4.5	4.6	4.5
**2**	4.1	4.1	4.1	4	4.1	4.3	4.1	4.2	4	4.2	4
**3**	4.3	4.3	4.2	4.4	4.3	4.3	4.6	4.2	4.3	4.6	4.2
**4**	4	4.1	4	3.8	3.9	4	3.9	4	3.9	4	3.9
**5**	4.3	4.2	4.4	4.3	4.4	4.2	4.2	4.3	4.3	4.4	4.3
**6**	4.2	4.3	4.1	4.1	4.2	4.2	4.3	4.3	4.1	4.3	4.3
**7**	4	4	4.2	4.1	4	4.1	4	4	4	3.9	3.9
**1**	4.3	4.5	4.3	4.5	4.2	4.4	4.5	4.5	4.3	4.2	4.3
**2**	4.1	3.9	4.1	4.1	4.2	4.2	4.1	4.2	4.1	4.2	4.3
**3**	3.9	3.9	3.8	3.9	4	4	4.1	3.9	3.9	3.8	4
**4**	3.9	3.9	3.8	4.1	4	4	3.8	3.9	4	3.8	3.9
**5**	4.1	4.1	4.3	4.1	4.3	4.4	3	4.2	4.2	4.3	4.2
**6**	4.1	4.1	4.1	4.1	4.2	4.2	4	4.1	3.9	4	4
**7**	3.9	3.9	3.8	3.9	3.7	3.9	3.9	4	3.8	4	4.1
**Total number of frames in 30 cycles**						1806					

		Recorded data									
**Observation | K_L=1mm**	**mean value**	**1**	**2**	**3**	**4**	**5**	**6**	**7**	**8**	**9**	**10**
**1**	5.1	5	4.9	5.1	5	5	5.2	5.2	5.5	5.1	5.1
**2**	4.4	4.5	4.1	4.3	4.5	4.5	4.4	4.5	4.6	4.4	4.2
**3**	3.8	3.7	3.8	3.9	3.8	3.9	3.8	3.7	3.8	3.5	3.8
**4**	3.5	3.8	3.2	3.5	3.8	3.4	3.4	3.3	3.4	3.5	3.5
**5**	3.8	4	3.8	3.9	3.6	3.4	3.8	3.6	4	4.1	3.7
**6**	4.3	4.5	4.5	4	4.3	4.3	4.4	4.4	4.4	4.3	4.2
**7**	4.5	4.6	4.7	4.5	4.5	4.2	4.6	4.3	4.8	4.6	4.5
**1**	5	4.9	4.5	4.8	5	5.5	5.1	5.1	4.8	5	5.2
**2**	4.5	4.4	4.9	4.3	4.4	4.5	4.1	4.6	4.5	4.5	4.7
**3**	4.3	4.1	4.3	4.6	4.1	4.3	4.5	3.9	4.4	4.5	4.3
**4**	3.3	3.4	3.4	3.1	3	3.3	3.5	3.4	3.5	3.1	3.4
**5**	4.3	4.4	4.2	4.4	4.4	4.5	4.5	4.2	4.1	4.3	4.1
**6**	4.5	4.8	4.7	4.4	4.5	4.2	4.6	4.6	4.7	4.2	4.6
**7**	4.9	4.7	4.8	5.1	5	4.9	5	5.2	4.8	4.6	4.7
**Total number of frames in 30 cycles**	1830										

		Recorded data									
**Observation | K_L=3mm**	**mean value**	**1**	**2**	**3**	**4**	**5**	**6**	**7**	**8**	**9**	**10**
**1**	5.2	5.1	5.3	5.1	5.2	5.5	5	5.1	5.2	5.2	5.4
**2**	4.4	4.4	4.3	4.4	4.2	4.5	4.5	4.4	4.4	4.1	4.4
**3**	3.7	3.6	3.4	3.7	3.6	3.7	3.9	3.8	4	3.6	3.5
**4**	3.3	3.3	3.5	3.6	3.3	3.2	3	3.4	3.3	3.1	3.2
**5**	4	3.6	4.1	3.9	4.3	3.7	4.2	4	4	4.1	4.2
**6**	4.5	4.3	4.8	4.2	4.3	4.4	4.6	4.3	4.6	4.6	4.5
**7**	4.9	4.5	4.6	4.9	5.1	5	4.7	5.3	4.9	5.1	4.8
**1**	5.3	5.1	5.4	5.5	5.7	5.2	5	5.6	5.3	5.1	5.2
**2**	4.2	4.2	4.2	4.5	3.7	4.1	4.7	4.1	4.1	4	4.4
**3**	3.2	3.1	3	3.4	3.3	3.3	3.3	3.2	3	3.3	3
**4**	2.9	2.7	2.7	3.1	3.1	2.5	2.9	3	2.8	3.1	3
**5**	3.3	3.2	3.4	3.2	3.4	3.3	3.1	3.5	3.4	3.6	3.1
**6**	4.7	4.6	4.4	4.7	4.7	4.6	4.8	4.7	4.7	4.7	4.8
**7**	5.2	5.1	4.9	5.2	5.2	5.3	5.2	5.1	5.3	5.2	5.4
**Total number of frames in 30 cycles**	1801										

		Recorded data									
**Observation | K_L=5mm**	**mean value**	**1**	**2**	**3**	**4**	**5**	**6**	**7**	**8**	**9**	**10**
**1**	5.9	5.9	5.9	5.9	5.7	5.7	5.7	6.3	5.8	6.1	6
**2**	5.5	5.4	5.5	5.6	5.6	5.5	5.5	5.4	5.6	5.4	5.6
**3**	3.8	3.9	3.6	3.5	4	3.8	3.9	3.6	4	3.4	4.1
**4**	3.1	3	3	3	2.9	3.1	3	3.4	3.1	3.3	3.1
**5**	3.4	3.5	3.2	3.3	3.2	3.7	3.5	3.3	3.2	3.4	3.7
**6**	4.6	4.7	4.7	4.4	4.6	4.8	4.3	4.8	5.1	4.3	4.2
**7**	5.7	5.7	5.9	5.7	5.7	5.7	5.5	5.4	6	5.8	5.5
**1**	5.8	6	5.5	5.8	5.9	5.6	5.7	5.8	6.2	5.9	5.7
**2**	4.5	4.6	4.6	4.9	4.3	4.4	4.2	4.7	4.5	4.4	4.2
**3**	3.7	3.5	3.8	3.8	3.7	3.8	3.7	3.7	3.6	3.9	3.7
**4**	3.1	2.5	3	3.1	3.2	3.4	2.9	3.3	3.1	3	3.1
**5**	3.6	3.6	3.6	3.6	3.5	3.4	3.8	3.6	3.9	3.4	3.5
**6**	4.3	4.4	4.1	4.7	3.9	4.2	4.5	4.1	4.3	4.4	4.5
**7**	4.9	5.1	4.9	4.7	4.8	5	4.4	5	5	5.1	5.3
**Total number of frames in 30 cycles**	1819										
